# “No decision about me without me” in the context of cancer multidisciplinary team meetings: a qualitative interview study

**DOI:** 10.1186/s12913-014-0488-2

**Published:** 2014-10-24

**Authors:** Cath Taylor, Jennifer Finnegan-John, James SA Green

**Affiliations:** Florence Nightingale Faculty of Nursing & Midwifery, Kings College London, London, UK; Breast Cancer Care, 5-13 Great Suffolk Street, London, UK; BartsHealth NHS Trust, Whipps Cross University Hospital, London, UK

**Keywords:** Cancer, Multi-disciplinary team, Clinical decision-making, Patient-centredness, Qualitative, Interviews

## Abstract

**Background:**

Cancer care is commonly managed by multidisciplinary teams (MDTs) who meet to discuss and agree treatment for individual patients. Patients do not attend MDT meetings but recommendations for treatments made in the meetings directly influence the decision-making process between patients and their responsible clinician. No research to-date has considered patient perspectives (or understanding) regarding MDTs or MDT meetings, though research has shown that failure to consider patient-based information can lead to recommendations that are inappropriate or unacceptable, and can consequently delay treatment.

**Methods:**

Semi-structured interviews were conducted with current cancer patients from one cancer centre who had either upper gastrointestinal or gynaecological cancer (n = 9) and with MDT members (n = 12) from the teams managing their care. Interview transcripts were analysed thematically using Framework approach. Key themes were identified and commonalities and discrepancies within and between individual transcripts and within and between patient and team member samples were identified and examined using the constant comparative method.

**Results:**

Patients had limited opportunities to input to or influence the decision-making process in MDT meetings. Key explanatory factors included that patients were given limited and inconsistent information about MDTs and MDT meetings, and that MDT members had variable definitions of patient-centredness in the context of MDTs and MDT meetings. Patients that had knowledge of medicine (through current/previous employment themselves or that of a close family member) appeared to have greater understanding and access to the MDT. Reassurance emerged as a ‘benefit’ of informing patients about MDTs and MDT meetings.

**Conclusions:**

There is a need to ensure MDT processes are both efficient and patient-centred. The operationalization of “No decision about me without me” in the context of MDT models of care – where patients are not present when recommendations for treatment are discussed - requires further consideration. Methods for ensuring that patients are actively integrated into the MDT processes are required to ensure patients have an informed choice regarding engagement, and to ensure recommendations are based on the best available patient-based and clinical evidence.

**Electronic supplementary material:**

The online version of this article (doi:10.1186/s12913-014-0488-2) contains supplementary material, which is available to authorized users.

## Background

Across much of Europe and many other countries worldwide cancer care is delivered by multidisciplinary teams (MDTs). In the UK cancer MDTs have a regular multidisciplinary team meetings (MDT meetings), similar to tumour boards, where relevant diagnostic, surgical, nursing and other healthcare professionals collaboratively discuss individual patients and agree recommendations for their treatment. Numerous observational and quasi-experimental studies have shown MDT working to be associated with a range of benefits including better clinical decision-making; clinical outcomes including survival; recruitment to clinical trials; and health professional satisfaction [1-5]. Patients do not attend MDT meetings but recommendations for treatments made in the meetings directly influence the decision-making process between patients and their responsible clinician.

Involving patients in decisions about their treatment is central to UK health policy and embedded in the National Health Service (NHS) Constitution, with the current government using the phrase “*No decision about me without me*” to describe their aspiration for the NHS [6]. In addition to an ethical rationale for patient involvement, other benefits may include better treatment adherence, reduced preference for major surgery, and more appropriate service use [7,8]. Effective involvement in decisions requires good communication between patients and health professionals. Interventions aimed at improving doctor-patient communication (via patient and/or health professional education) have been associated with a range of positive health outcomes for patients including improved functional status, symptom resolution and decreased anxiety [9,10].

The terms ‘patient-centred care’ and ‘shared decision-making’ are used liberally but lack agreed definitions [11]. Furthermore evidence suggests clinicians are poor at judging patients’ preferences for treatment [12] and may have different goals to patients with regard to shared decision-making. In a study involving 70 disease-free rectal cancer patients and their clinicians, almost all clinicians defined patient participation as ‘reaching an agreement’ whereas almost a quarter of the patients defined participation as simply ‘being informed’ [13]. A Canadian national population-based study showed that preferences for participation in decision-making may vary considerably [14]. Although nearly all reported that they wanted to be informed and offered choices, half preferred to leave final decisions to their responsible clinician. Being female and more educated was associated with preferring more active involvement (measured in relation to knowledge-seeking, desire for options and involvement in decisions).

The results from the 2011/12 UK National Cancer Patient Experience Survey [15], completed by over 70,000 cancer patients, highlighted variable practice across different hospitals. Between 62-100% of patients reported being given a choice of treatments prior to treatment commencing, and between 56-83% of patients reported that their views were taken account of by their team. Nearly one in ten patients (8%) reported they did not even know their treatment was discussed by a team.

No research to-date has considered patient perspectives (or understanding) regarding MDTs or MDT meetings, or how this model of working impacts on patients’ involvement in the decision-making process. However, a systematic review highlighted that one of the key barriers to effective decision making in MDT meetings was lack of consideration of patient-centred information [16]. Where such information is not at the heart of decision-making in MDT meetings there is a risk of poor quality clinical decisions. These may be unacceptable to the patient and/or not clinically appropriate, which may lead to the need for re-discussion by the MDT and consequently may delay treatment [17-20].

This study aimed:

- To explore what current cancer patients know, and need to know, about the MDTs involved in their care to ensure effective involvement in decision-making.
- To examine cancer MDT members’ views on how best to ensure that patients are meaningfully involved in decision-making about their care in the context of the MDT and MDT meetings.
- To gain patient and MDT members views on how to overcome any barriers identified and improve current practice.

### Design and sample

Semi-structured interviews were conducted with current upper gastrointestinal (upper GI) and gynaecological (gynae) cancer patients from one cancer centre (a hospital providing specialist cancer services), and with MDT members from the teams managing their care. Both tumour types are relatively common and include a sizeable proportion with advanced or metastatic disease, which in some cases require complex treatment decision-making. Also, both tumour groups fared relatively poorly with regard to involvement in decisions in the 2010 national patient experience survey, in which over a third of patients treated by these teams did not feel involved in decisions about treatment [21].

## Methods

Patients were screened for eligibility by a clinical staff member and their eligibility confirmed by the patient’s relevant Clinical Nurse Specialist (CNS) using inclusion and exclusion criteria (Table 1). Eligible patients were recruited through a two-stage consent process, first being approached by their CNS to gain consent to share their contact details with the research team; and, if consent was received for this, the researcher sent a detailed study information sheet and consent form with a stamped addressed envelope. Interviews were arranged with all consenting patients. We recruited all eligible and consenting patients between July-October 2012, (n = 9: 4 = gynae, 5 = upper GI). Patients were offered either face to face or telephone interviews (all opted for telephone interviews).

**Table 1:** **Inclusion and exclusion criteria**

**Inclusion criteria**	**Exclusion criteria**
Patients	Patients
• Current out-patients who have completed primary treatment for Upper GI or Gynaecological cancer within the previous two months	• Aged 17 or under
	• Unable to speak English
	• Unable to cope either physically or emotionally, with the research protocol
• Able to give informed consent	• Patients with major communication or cognitive problems (i.e. degenerative illness or other condition affecting cognition and/or comprehension).
• Sufficient understanding of written and spoken English to enable participation	
MDT members	
• Core MDT member	
• Responsible for communication about treatment (including Oncologists, Surgeons and Clinical Nurse Specialists)	

All core MDT members that had responsibility for communicating with patients about treatment, were emailed an invitation to participate. This included all surgeons, oncologists and CNS’s. Non-responders were sent a second invite. In total 12 of the 20 eligible MDT members participated in an interview (5 = gynae; 7 = upper GI). Interviews were conducted either face to face (n = 10), or by telephone (n = 2).

Interviews were semi-structured, and were predominantly exploratory in nature though some descriptive data was also collected (e.g. whether or not patients had heard of an MDT or MDT meeting, or knew their case was discussed by a group of professionals). All interviews lasted 30-45mins and followed a topic guide based upon the study aims (see Additional file 1).

The protocol for this study was reviewed and granted approval by NRES Committee East Midlands – Leicester (Reference: 12/EM/0166).

### Data analysis

Interviews were recorded digitally, transcribed and analysed using the “Framework” approach [22]. Framework is a matrix-based approach for collating, reviewing and understanding qualitative data. Two experienced qualitative researchers (JFJ and CT) independently read a sample of transcripts and discussed and agreed key themes. Due to the a-priori interest in comparing patient and team member’s views, two separate thematic frameworks were created to facilitate examination of similarities and divergence between and within patients and staff members, using the constant comparative method. In order to maintain anonymity, all transcripts were given unique ID pseudonyms. Quotations from patients are attributed to tumour type, whilst staff are attributed to Team A or B.

## Results

### Characteristics of participants

Twenty-one interviews were conducted. This included nine patients aged 25–80 years, most from White British ethnic origin (Table 2). At the time of interview, two Upper GI patients were awaiting further surgery and all others had completed treatment. Twelve MDT members were interviewed including the MDT lead and two CNS’ from each team together with other medical MDT members (oncologists and surgeons, Table 3).

**Table 2:** **Characteristics of patients**

**Tumour type**	**Total number of interviews conducted**	**Characteristics of patients**		
		**Gender**	**Age**	**Ethnicity**
**Gynaecological**	4	Female	68 years	White British
		Female	61 years	White British
		Female	52 years	White, Australian
		Female	25 years	White British
**Upper GI**	5	Male	80 years	White British
		Male	75 years	Black Caribbean
		Male	48 years	White British
		Female	74 years	White British
		Female	61 years	White British

**Table 3:** **Characteristics of MDT members**

**Tumour type**	**Total number of interviews conducted**	**MDT members’ specialty/role in the team**
**Gynaecological**	5	MDT Lead (Gynaecologist)
		2 x Clinical Oncologists
		2 x Clinical Nurse Specialists (CNS)
**Upper GI**	7	MDT Lead (Surgeon)
		2 x Clinical Oncologist
		1 x Medical Oncologist
		1 x Surgeon
		2 x CNS

Findings indicated that patients had limited opportunities to input to or influence the decision-making process in MDT meetings. Key potential explanatory factors that emerged from the thematic analysis include:

1. Limited and inconsistent information provision about MDTs and MDT meetings
2. Variable definitions of patient-centeredness (in the context of MDTs and MDMs) by MDT members
3. The potential influence of patient knowledge about medicine on their understanding (or recall) of information about MDTs and MDMs

### Limited and inconsistent information provision

i) What are patients told about the MDT and the MDM?

Most MDT members acknowledged that patients probably had a limited understanding about MDT/MDM’s and that at most they might understand that recommendations for treatment were the result of an MDT discussion between groups of different health professionals: “*a panel of experts*” (Dr Jones, Team B). *"I think they understand that we meet and discuss cases but … I’m not sure they understand the number of people involved or exactly what we do"* (Dr Smith, Team A).

Indeed a few team members described what they said to patients about the MDT/MDM. In these examples the information given was sparse and described patient’s as being passive receivers of information, rather than explicitly inviting their contribution and involvement: *"what I say to patients about the MDT, so quite often when there’s a difficult decision or when the treatment options aren’t clear I do say to the patients we will be discussing your case on Wednesday and then we’ll make a decision about what we think is the best treatment for you"* (Dr London, Team A). Another team member stated *"I think every one of them that have scans done as part of their investigation they usually get told that … it’ll be discussed in the meeting”* (Nurse Roberts, Team A).

Although most team members stated that they at least mentioned the MDT/MDM to patients, one doctor stated that patients would not be interested in knowing details about the MDT/ MDM:

*"The MDT is there not to be communicated to the patient but just to make the process safe … why does the patient have to know this? It’s just a complication, it’s another thing for the patient to take-in in a clinic where they are being told all sorts of things and it’s just another distraction and I’m not sure it’s of any relevance"* (Dr Drew, Team B)*.* A similar view was shared by another doctor from Team B who said he reserved information for those who asked rather than offering it to all patients:

*“Patients understand that their condition will be discussed by a panel of experts … they know those individuals will be a mixture of surgeons and oncologists, but we don’t go into greater details than that unless they ask … they’re interested in what the treatment options are and what you’re going to do for them. They’re not as interested in the process … they’re interested in outcome”* (Dr Jones, Team B).

ii) How are patients informed about the MDT/MDM?

MDT member’s knowledge of information resources they could use to explain the role and membership of the MDT to patients was variable. One doctor from Team A mentioned a DVD resource, but nobody else from either team mentioned this. In total, four members (2 from each team; doctors and nurses) mentioned providing patients with a booklet, but other members within the same teams did not mention using this resource, although most were aware that this was available. The information within these booklets varied by tumour type but at most included the team member’s names and contact details. The majority of team members (n = 8) did not mention using any resources in their interaction with patients; most of whom (n = 6) reported that information about the MDT and MDM was generally provided verbally; the others (n = 2) stated they generally did not provide any information, believing it to be unnecessary.

Narratives from patients reflected this unsystematic approach to information provision. When asked what they understood about the MDT and MDM, three patients reported that they had not been told anything – at least not at the time their treatment was being planned. One patient stated that she was not told about the MDM until she was undergoing treatment: *“I think I was on my 2nd chemotherapy”* (Sarah, Upper GI patient)*.* Three patients discussed being given information about the MDT and MDM verbally by their CNS, none of whom recalled being given a booklet or any other resource. No patients recalled being given a DVD and none specifically mentioned reading about the MDT in a booklet. One patient raised the benefits of having written information: *“I think all information should be written that they’re giving because when you are that stressed you don’t take things in, you might take somebody with you but they might not take it in either … at least you’ve got it in writing if you want to know … you know you’ve got a piece of paper that says something somewhere”* (Mary, Gynaecological cancer patient).

Some of the MDT members explicitly recognised that patients were often overwhelmed with information at the point of diagnosis (the point when they commonly come into contact with their MDT). They queried how much information patients would be able to absorb. A nurse from Team B stated: “*They actually have a little leaflet to try to explain [the MDT] but… they get leaflets on just about everything else so that doesn’t always get read and absorbed”* (Nurse Brown, Team B)*.* A doctor from the same team concurred: *“patients have an increasing amount of information to take in and when they come to the clinic quite often they are literally burdened with just reams and reams of pieces of paper … they just can’t take it in and also the amount of information that a patient can take in at any particular consultation is very limited … [They] tend to remember the important thing you say to them at the beginning and … the last thing that was said to them before they leave the room. For that reason it’s quite limited in terms of telling them about [the MDT] process that sort of obviously directly applies to them but they don’t necessarily have much control over, and the consultations tend to be very factual telling them about the treatment and why they are having that treatment and going through the rationale and side effects and practicalities and all that sort of thing”* (Dr Jones, Team B)*.*

When patients were able to recall who had told them about the MDT/MDM they all reported that it was their CNS. Four members of Team B (both CNS’ and 2 doctors) described the importance of the CNS in relation to this aspect of information-giving due to their level of contact with the patient. Members of Team A generally expressed the view that whoever saw the patient would inform them about the MDT and MDM, rather than it being a specific responsibility for CNS’s (or any other team member).

### Variable definitions of patient-centeredness

MDT members had differing views of what “patient involvement” meant within the context of the MDM and the treatment decision-making process. Some clearly articulated the value of the patient being informed and actively involved (if they wanted to be), whereas other team members (as mentioned previously) had a view that MDTs and MDMs were about ‘organisation’ or ‘process’ and not of relevance to patients. Almost all team members described shared decision-making with patients taking place *after* the MDM (when treatment choices may be presented to patients). Although most team members recognised the value and importance of having patient-centred information shared in the MDM, this was not described or acknowledged as being part of a shared decision-making process and was instead focussed on collation of information from patients.

i) Defining patient-centredness as providing treatment choices after the MDM

Team members who perceived that patients were adequately involved in the treatment decision-making process often reasoned that this was because patients had a choice in their treatment and were given the option to agree or disagree with MDT recommendations: *"Well they are … very effectively involved in the decision making aren’t they, we offer treatment … and suggest treatment, so they are involved in their care … and we alter treatment according to patient’s wishes … I say this is what I think is the best treatment for you and if they ask me whether there are other treatment options available I would suggest it, if those options were sensible”* (Dr Peters, Team B)*.*

One nurse mentioned that patients were given a choice but the specific example she gave was of a situation where the patient was given choices only after refusing the recommended option: *“You have the odd patient who says ‘no way - I’m not having surgery’ so we explain to them that’s fine, the other option is chemo and radiotherapy or just radiotherapy or just chemotherapy, we will get you to have a chat with them, that consultant, that speciality and see what they say"* (Nurse Brown, Team B).

Team members had differing views regarding the issue of whether patients wanted choice. Some team members recognised that patients felt overwhelmed by being given choice about treatment: “*A lot of patients don’t want to be given a vast amount of choice – they come to see you as a specialist because you have expertise in the area, and they hope you will guide them into what are the best options. But of course when they ask us questions we are always very open and encourage them to ask questions"* (Dr Jones, Team B). In contrast another doctor from Team B expressed his view that their practice was not as patient-centred as he would like and that providing choices would improve this: *"I think maybe* [we need to] *give options … we recommend either treatment plan A if the patient wants very radical treatment and really wants to go for it or treatment plan B. We could do that a little bit more. We tend to recommend one option rather than two or three different options"* (Dr Simons, Team B).

Most patients were satisfied to leave decision making about treatment to their doctor: *“I’m happy to sort of just go along with what’s happening, if you get what I mean”* (Christopher, Upper GI patient).

Several patients specifically commented that they were not in a position to tell the team what they would want to happen regarding their treatment. These patients believed that it was better to trust the expertise of the team: *“You are not going to turn around unless you are a doctor or a nurse or someone who knows about medical things and argue with them. That was their decision, they said it was the best decision and that’s the decision they need to take to make sure that you are going to live basically. Your life is in their hands”* (Jane, Gynaecological cancer patient). One patient understood she only had one option for treatment: *"The thing is in my case there wasn’t any other options, it was either I had treatment or I didn’t have treatment and that was the form of treatment that they recommended but I could have refused it obviously"* (Mary, Gynaecological cancer patient).

Only one patient specifically mentioned discussing treatment choice before the MDM. She had a preference regarding the type of incision for surgery which she communicated to the MDT via her CNS, who shared it the MDM. The MDT recommendation differed from her preferred option due to clinical reasons but despite this she felt that the recommendation was for her benefit, and that she had been actively involved in the process: *"I don’t have enough knowledge to make these decisions for myself,* [I would have preferred to] *have a horizontal* [incision] *though it may not have been* [beneficial] *for me, [so] I would prefer that decision be made on my behalf"* (Lauren, Gynaecological cancer patient). She went on to explain that it was important that patients were made aware why particular recommendations were made by the MDT: *“if you are given enough information to fully understand why it is they’ve made this decision, the things that they’ve thought about and dismissed… then I think it’s best to know those sort of things”.*

ii) The collection and use of patient-centred information

Differing views and practices existed in relation to the collection and use of patient-centred information. One team member expressed particular dissatisfaction regarding the collection and/or sharing of such information, when occasionally some patients may be presented at the MDM without team members having any prior knowledge about them: *"I mean there are certain recommendations that you should make, I mean they’re common sense recommendations anyway which is firstly that somebody who knows the patient well is available at the MDT to present the case and knows all the various social circumstances, who knows the family situation, who knows what the patient’s wishes are because if that information is not available … we can make a decision based on the science, we can make a decision based on the imaging but that’s not a patient-centred decision … for me the whole purpose of getting everyone together is because we’re not robots and because patients do matter and they’ve all got, they do have their opinions and they’ve all got very different social, economic circumstances and different religions”* (Dr Simons, Team B).

A nurse from Team A felt that the quality of patient-centred information was related to the professional group of the team member preparing the case for discussion: *“I think that probably in most patients you could do with more information on their social situations but I think that is to do with whoever fills out the form and my bias would be that the nurse specialist who fill them in probably put in quite a bit of information as usually is our nature and I would say that maybe junior registrars or junior doctors may put in what they see as essential”* (Nurse Roberts, Team A).

Most of the members of Team B stated that collection of patient-centred information was a particular responsibility of the CNS and recognised the value of having such information when making treatment decisions: *“a lot of the nurse specialists that report in, some of them are extremely good at making those assessments and they will often say to us that patients and their relatives don’t want any active treatment, we’ve spoken to them about it and they’re quite happy to have palliative treatment, and that’s fine. We don’t argue any further, and the nurse specialists on the whole will act as an advocate. It sounds rather judicial, but on the basis on the patient’s wishes, we do that”* (Dr Jones, Team B)*.*

Within Team A, both CNS’ discussed how they saw it a part of their role to collect patient-centred information and present it at the MDM: “*I always feel as a CNS … this is a really big part of my role to get the patient’s wishes over because … I see that as a form of support"* (Nurse Roberts, Team A). However, other team members reported that collection and presentation of such information in the MDM should be the responsibility of whoever refers the patient to the MDT: *"it’s very much the person that refers the patient to the MDT what they’ve put on the form or what they say in the meeting and that’s why it’s so important that someone is there to know what the patient, to represent those patients wishes"* (Dr London, Team A)*.*

Two patients (both retired healthcare professionals) recalled being asked specific questions about themselves which they assumed would inform decision-making about their treatment. James stated: *"Oh yes all the time I’ve been asked these questions – who’s at home, who looks after you, what will things be like when you are at home and also my previous medical history … I think they might influence what decision the MDT comes to but again that’s entirely what you’d expect them to do having the information made available to them"* (James, Upper GI cancer patient)*.* Celia described how her living conditions and caring responsibilities might impact on the treatment decisions made: *"in my case it was important because* [I live in] *a house with stairs and steps and complications and an elderly husband who partly needs looking after. So there was quite a bit of that being thought about” (Celia, Upper GI patient).*

Several patients (all without knowledge of medicine) recalled their discussions with team members to be solely clinically or tumour-focussed, rather than person-focussed. Catherine stated: *"No they didn’t really ask me anything about my life, didn’t ask about children if I remember, the only thing, at the initial interview I had with one of the team when I was told that this is what’s going to happen … and I said ‘oh, well I’m a carer for my Mum’ and they said ‘you’ll have to sort that out’, that sort of attitude, you can’t do it … have you got somebody in mind, and I said ‘oh yes I’ve got sisters, I’ll sort that out’”* (Catherine, Gynaecological cancer patient)*.*

iii) The consequence of incomplete patient-centred information

One doctor from Team B felt that patient-centred data had no place within the MDM, as the MDM was a “*technical discussion”*. He felt that the clinic was the place to make a patient-centred decision, once the recommendation had been presented to the patient: *"I don’t personally think the [MDM] is the place to bring it up because a number of reasons … the religious and cultural beliefs of the person without the person themselves are a) most likely to be misrepresented, b) pretty much meaningless without the human being there … you’re not managing the patient in an [MDM], you are just making a decision on part of the information and it’s a part of the information that needs to be analysed as a group because ideally you want different people to look at the same information and come to a collective decision but you don’t need them to come to a collective decision on how this impacts on cultural beliefs”* (Dr Drew, Team B).

In contrast, other team members described the value of having patient-centred information in the MDM, particularly in relation to tailoring treatment recommendations. A doctor from Team B stated that if patient-centred information was not available in the MDM the recommendations they make for treatment might not be appropriate: *“If nobody in the MDT knows the patient or the notes are not available it’s more than likely that the treatment plan that’s recommended is not going to be the correct treatment plan, so MDT’s can make mistakes. The MDT decision is not necessarily the correct one”* (Dr Simons, Team B).

Another doctor from Team B felt that collection and presentation of even the smallest amount of anecdotal information about a patient had value and really helped to bring the case discussion “to life” within the MDM and enabled them to personalise treatment decisions: *"I think what is very useful is some people will even use an anecdote of what the patient said and so forth to make the person almost appear at the MDM meeting. You don’t need to have a two page description but if you say this is a 72 year old bus driver … give background information on the patient and then you can start picturing the patient because everybody has got experience in the MDM … So we tailor our treatment a little bit to who we are dealing with"* (Dr Reed, Team B).

A nurse from Team A described how information about a patient’s circumstances, particularly any co-morbidities, could be used in the MDM: *"so lots of us will be very interested in their social situation … we nearly always know if they have dementia and whether they have capacity to make decisions themselves or whether a relative is helping make the decisions or whether they’ve no-one at all to make the decisions and then we need to get an advocate in to help them to make the decision”* (Nurse Roberts, Team A). A doctor from Team A acknowledged that some clinicians discount personal circumstances from the decision-making process in MDMs but argued against this: *“We would say at the MDM like certain things like age and sex and religion and all these things doesn’t influence the decision but it does sometimes because if you are 85, you know, and you are a Jehovah’s Witness who won’t have a blood transfusion … you might just think twice about surgery”* (Dr Reed, Team, B).

Both MDTs were specialist MDTs and therefore received referrals for treatment recommendations from teams in peripheral hospital sites. Several members from both teams stated that decision-making was compromised when the peripheral team omitted patient-centred information: *"We have some Trusts that don’t link in regularly and therefore we’re discussing their patients without any knowledge at all of the patient … we’re making decisions based on limited information … one of the risks of that is you could be making a recommendation that is completely inappropriate and you assume hopefully that someone who actually sees the patient will take the MDT decision as it should be which is a recommendation, but not as written in stone"* (Dr Cox, Team B).

A nurse from Team A concurred with this and stated that the process of collecting information about patients prior to MDMs could be improved *"…[the local team] could probably say to [patients] ‘you will at some stage be discussed in our multidisciplinary team meeting and you may be discussed several times – is there anything you would like [specialist team] to know about you or is there any, have you strong religious beliefs, have you strong wishes that you want them to adhere to or, you know?’, so I suppose there could be that kind of thing”* (Nurse Roberts, Team A).

### The potential influence of patient knowledge about medicine on their understanding (or recall) of information about MDTs and MDMs

A potential explanatory factor that distinguished those with better vs. worse understanding about MDTs/MDMs was the presence or absence of a link to medicine (e.g. through family or their own professional background). Three patients had such links to medicine (two were retired healthcare professionals themselves - doctor and a physiotherapist - and the other had a father and fiancé who were doctors) and all were able to articulate clearly their understanding of the function and membership of the MDT. For example, Lauren stated: *"When it was initially thought that my MRI was suspicious I was told that they would be having a multidisciplinary meeting regarding me to decide the best course of action so I had been told that this was going to happen … they did explain to me in the meeting that these were the sort of people who were going to be making decisions on me. It was quite clearly explained to me before the meeting took place … I was quite well informed from that point of view … my understanding was that it was a team of specialists from differing branches of medicine, the radiologist who would have a look at my MRI and see exactly what he thought of it and describe it to the surgeon … and [involve] other specialists in that area. [This] was my understanding of the team”* (Lauren, Gynaecological cancer patient). Lauren was also the only patient that communicated preferences for treatment to the MDT prior to their MDT meeting.

In contrast, patients without such links to medicine demonstrated a much vaguer (if any) understanding of the MDT and MDMs*: “[I understood] very little really [about the MDM] only that they would look at my case and they would say what was the best course of action to go … I didn’t know who could be involved apart from the surgeon”* (Catherine, Gynaecological cancer patient).

### Reassurance as a benefit of informing patients about MDTs and MDMs

“Reassurance” was a recurring theme in some MDT member and patient transcripts. One doctor from Team A alluded to the importance of informing patients about the MDT because it would be reassuring to know that their treatment plan arose from a team discussion: *"Well I mean I think the more information they have about our processes the better really. I don’t think there’s anything wrong about them knowing what we do, we don’t do anything wrong, it’s all in their benefit so no I think it would be good information to provide in that we have MDM’s and all the patients are discussed … I think it would be good that they know that that happens”* (Dr Smith, Team A). A nurse from the same team shared this view about the benefit of ensuring patients understood the process: *"I think they would feel more empowered if they thought there were a lot of people involved in the decision"* (Nurse Roberts, Team A).

Some patients’ narratives concurred with this. Catherine, despite stating that she did not know anything much about the meeting or who the team were stated: *"They said they will look at all the scans and they will assess the situation … so I did know there was a team looking at it. And I was reassured because obviously they do this all the time, and they would know"* (Catherine, Gynaecological cancer patient)*.* Another (a retired physiotherapist) stated: *“A multidisciplinary team is always a good thing, it’s amazing, it makes a hell of a difference to have access to the other disciplines straight away so it would have to be a good thing"* (Celia, Gynaecological cancer patient).

A nurse from Team A discussed how patients would be reassured to know that someone they had already met would be involved in making a treatment decision: *"They sometimes want to know who is going to be there [at the MDM] so if there is a particular surgeon that they knew from before, and we’re talking about maybe possibility of surgery they will ask ‘is my surgeon going to be there?’ , and actually I quite often volunteer that information if I’m feeding back from what we discussed"* (Nurse Grant, Team A). One patient described the impact of having this information; she was reassured by knowing which team member was involved in making the decision on her behalf: *“He was the one face that you saw that you knew he was in control of it all”* (Jane, Gynaecological cancer patient).

### Team members and patients recommendations for changes to current practice

Recommendations for changes to current practice were suggested by team members and patients. These recommendations focused on improving communication and information provision, and collection and use of patient-centred information. Team members’ recommendations for improved communication ranged from inserting a simple sentence in discussions: “*something should be in the guidelines when we give people information about their treatment, to the effect that all treatment decisions will be made by multidisciplinary team review but I don’t think it needs any more than that really"* (Dr Peters, Team A), through to a focus on encouraging patients to be actively involved in decision-making, though still with a focus on post-MDM: *"The MDT will decide on this or this on your behalf and you of course have an option to come back on that again and say that I’m not happy with the MDM decision and maybe the patient should be informed that they can possibly disagree with the MDM decision and say I don’t want the MDM to decide, I want to decide that and so the MDM can be advising me but not deciding on my behalf”* (Dr Reed, Team B).

Team members also shared recommendations for improving the collection and use of patient-centred information. One stated that there should always be someone present in the MDM that has met the patient where possible; and another suggested that a minimum dataset that includes patient-centred information should be used (especially from peripheral teams): *"what we’re trying to do is say look, unless you fill in the minimal dataset which will also include things like that, and co-morbidities, other medical issues … we won’t discuss the patient. The problem is obviously ethically it becomes very difficult if a patient’s very ill and needs treatment, and you’re saying, you haven’t ticked this box so we’re not discussing it, it gets a bit tricky”* (Nurse Grant, Team B).

Recommendations from patients included a focus on actively involving patients prior to the MDM by providing an easy way to communicate their preferences or wishes regarding treatment to an advocate prior to MDM. One patient suggested that patients could be given a form to complete: “*a form or something like ‘do you have any questions for the team?’ or…‘anything you would like to be brought up on your behalf?’… maybe where [patients] could just get what they want to say across and be sure that it was relayed and spoken on their behalf”* (Lauren, Gynaecological cancer patient). one patient suggested that they should have an opportunity to ask questions about their treatment prior to treatment commencing, suggesting this had not happened in her case: *"Once they made a decision on what they’re going to do with you then I think that’s a good opportunity to sit down with the patient again and say this is what we’re going to do… it’s probably good to sit down then and tell you exactly what you’re going to have before you go into the major surgery just to ask a few more questions like do you really need to take my ovaries, what are the reasons why you have to take them. There could be another opportunity there to make sure that the patient has answered as many, all the questions that they need before the surgery"* (Jane, Gynaecological cancer patient).

## Discussion

Patients had limited opportunities to input to or influence the decision-making process in MDT meetings. This appeared to be explained at least in part by a combination of limited and inconsistent information provision, and variable attitudes of health professionals regarding the importance of considering patient-centred information in MDT meetings.

To our knowledge there has been no previous research that has examined patients’ knowledge and understanding of MDTs or MDT meetings. The qualitative design of this study was not intended to produce generalizable findings but instead to examine in detail the viewpoints of patients and health professionals in a small number of teams in order to generate understanding and hypotheses that could be further tested in subsequent research. Resources limited the length of time for recruitment of patients: interviews with a broader spectrum of patients in relation to gender, age, ethnicity and disease status would have been beneficial and should be a focus for future research.

Some of the key findings that warrant further examination include first, the potential influence of prior knowledge of medicine. Previous research has shown that demographic characteristics such as gender (being female) and education (being more highly educated), as well as disease severity influence the extent that patients desire active involvement in decisions about their care [14,23]. The educational status of patients is unknown for the participants in this study but it may be that the finding regarding knowledge of medicine is instead explained by education status. Also we cannot know whether those that were able to articulate a clearer understanding of MDTs and MDT meetings were provided with more information than other patients (for example perhaps by asking more questions), and/or were able to understand or recall it more easily than other patients. Unpicking this would require an ethnographic observational approach where both information provision and understanding are assessed.

Second, this study highlighted that health professionals have wide ranging views regarding the patient-centredness of the decision-making process in the context of MDTs and MDT meetings. This directly influenced the communication they had with patients about MDTs/MDMs. In view of the impact of lack of patient-centred information on implementation of recommendations [17-19] and in line with the aspiration of the current government for ‘*no decision about me without me*’ it is important to address this and ensure – where possible –that patients are informed about MDTs in a way that allows them to actively engage with the decision-making process should they wish to. The public inquiries following significant failures of care within the NHS in recent years (e.g. in Alder Hey [24] and Mid Staffordshire [25]) have reinforced this imperative, and the need to embed shared decision-making into the NHS as opposed to passive consent [26]. Shared-decision making in the context of MDTs requires careful consideration. A survey of over 2000 cancer health professionals in the UK found that the majority felt it was neither desirable nor practical to include patients in MDT meetings [27]. Typically, each patient is only discussed for a few minutes (a colorectal cancer study reported 4.5mins average [28]), requires frank and disinhibited consideration of treatment options, and uses medical and technical terminology that would be at best incomprehensible, and at worst frightening to many patients. This does not however preclude ensuring that patients are adequately informed about the way that decisions or recommendations will be made and given an opportunity to engage in the process, e.g. by providing any information they wish to be taken into account, should they wish to.

There are a number of challenges associated with this, not least the pressures of adhering to national targets for reduced waiting times (between diagnosis and treatment) meaning that MDT discussions may often happen before patients are even aware of their diagnosis, let alone had time to consider their preferences or factors they would want taken into account when treatments are considered. Undertaking holistic needs assessments [29] at appropriate time points along the pathway of care, and ensuring that sharing of such information is prioritised in MDT meeting discussions is likely to improve the patient-centredness of the decision-making process. The CNS has a major role in relation to this as they will often be the team member assessing the holistic needs and acting as the patient’s keyworker [30]. However, research has shown that nurses may often contribute minimally in MDT meetings [31], which may at least in part be due to prioritisation by the medical team members of a disease-focused rather than person-focused approach [20].

In addition to ensuring effective mechanisms for obtaining information from patients, there is a need to develop effective ways of supporting patients to make informed decisions about participating in decision-making. In this study we found that information provision about MDTs and MDT meetings was limited and inconsistent. Collaborative design methodologies such as Experience Co-Based Design [32] could be used to work with patients and staff to design and develop information aids and interventions (e.g. patient and health professional education) to ensure effective communication between health professionals and patients regarding the way decisions are made. Patient decision aids have been developed and shown to be effective at improving knowledge and patient involvement in other health contexts, including prostate cancer screening [8,33] and should be examined for application in the context of the MDT.

A particular issue regarding patient-centredness in decision-making by specialist MDTs was raised in this study. Guidelines for best practice support recommend that MDT discussions always include at least one person that has met the patient [34]. The barriers and facilitators for ensuring that this can happen, especially in exchanges between local and specialist teams, should be urgently examined. Patients felt reassured when they knew that decisions were made by collectively by a team, and in particular when they knew their clinician would be part of that team; a finding also echoed in a recent study with prostate patients [35].

## Conclusions

Patients had limited knowledge of MDTs or opportunities to input to MDT meeting discussions. Advances in knowledge about cancer have led to increasing complexity with regard to diagnosis and treatment of the disease, and thus reinforce the imperative for a multidisciplinary approach to its management. There is a need to ensure MDT processes are both efficient and patient-centred. The operationalization of “No decision about me without me” in the context of MDT models of care requires further consideration. Methods for ensuring that patients are actively integrated into the MDT processes are required to ensure patients have an informed choice regarding engagement, and to ensure recommendations are based on the best available patient-based and clinical evidence.

## Additional file

Additional file 1:
**Topic Guide for interviews.**
