# Supplementary material for: “No decision about me without me” in the context of cancer multidisciplinary team meetings: a qualitative interview study
Source: BMC Health Serv Res. 2014 Oct 24;14:488. doi: 10.1186/s12913-014-0488-2 (PMC4210563; doi:10.1186/s12913-014-0488-2)
Supplement: Additional file 1: — Topic Guide for interviews. [file 12913_2014_488_MOESM1_ESM.docx]

**Taylor, Finnegan-John, Green: Study Topic Guide**

**Short study title: What do cancer patients know, and need to know, about their MDT?**

***Cancer patients:***

**Introduction**

This study aims to explore your views, experiences and knowledge of the multidisciplinary team of doctors and nurses that make recommendations about your treatment. Are you aware that the recommendations for your cancer treatment came from discussions held in an MDT meeting? *If yes, proceed; if no, explain about the MDT and MDT meetings.*

Topics to explore:

- Understanding about the membership and function of the MDT managing their cancer care
- Understanding about MDT meetings (the role/purpose, who attends etc)
- How involved they felt in decisions about their treatment (describe, needs/preferences taken into account? Timing of involvement in relation to MDT meeting?)
- Does communication about MDTs and MDT meetings need to be improved? How?
- Does the process of treatment planning involve patients sufficiently? (if not, how can this be improved?)

***Team members***

Topics to explore:

- Communication of information about MDTs and MDT meetings to patients (written/verbal), is it understood/sufficient?
- What they think patients need to know about the MDT/MDT meetings in order to be effectively involved in decision-making about their care
- Patient-centredness of decision-making in MDT meetings – could it be improved? What are the barriers and how might these be overcome?
- Does communication about MDTs and MDT meetings need to be improved? How?
- Does the process of treatment planning involve patients sufficiently? If not, how can this be improved?
